# Clonal Comparison of *Staphylococcus aureus* Isolates from Healthy Pig Farmers, Human Controls, and Pigs

**DOI:** 10.3201/eid1105.040866

**Published:** 2005-05

**Authors:** Laurence Armand-Lefevre, Raymond Ruimy, Antoine Andremont

**Affiliations:** * Groupe Hospitalier Bichat-Claude Bernard, Paris, France

**Keywords:** S. aureus, Pig farmers, MLST, Phylogenetic distribution

## Abstract

Pig farming is a risk factor for increased nasal *Staphylococcus aureus* colonization. Using sequence typing and phylogenetic comparisons, we showed that overcolonization of farmers was caused by a few bacterial strains that were not present in nonfarmers but often caused swine infections. This finding suggests a high rate of strain exchange between pigs and farmers.

Pig farmers work in close contact with animals that are given heavy loads of antimicrobial agents and therefore are highly colonized by resistant bacteria (1). The transfer of resistant bacteria from farm animals to farmers has been demonstrated in several instances (2,3). In a recent comparison of pig farmers and nonfarming controls, farmers were at a significantly greater risk for colonization by resistant commensal bacteria, including fecal enterobacteria and enterococci, nongroupable throat streptococci, and nasal *Staphylococcus aureus* (4). The rate of nasal *S. aureus* colonization was also significantly higher in farmers, in whom it reached 44.6%, compared to 24.1% in controls (4). The latter rate was similar to that observed in published cross-sectional prevalence studies conducted among study participants living in healthy communities (5). However in the previous study, we did not investigate the sources and origin of nasal *S. aureus* colonization and resistance in farmers. Here, we used the gene-based, recently developed technique of multilocus sequence typing (MLST) (available from www.mlst.net) to describe the characteristics of these nasal *S. aureus* strains from farmers and controls and the relationships between strains, and investigated their possible animal origin by comparing them with strains isolated from infected pigs from the same geographic area.

## The Study

The *S. aureus* strains studied included 44 nasal isolates from healthy pig farmers and 21 from healthy nonfarmer controls (i.e., bank or insurance workers). These participants all had been part of the population included in a previously published epidemiologic study in which the resistance rates in commensal bacteria from healthy pig farmers were compared with the rates in controls matched for age, sex, and county of residence (4). This population was disseminated over 7 French departments, chosen because they were the leading areas of porcine production. A department is a French administrative territory roughly the size of a British or American county. Each pig farmer worked on a different pig farm. We also studied 14 *S. aureus* isolates from the following types of swine infections: cutaneous, for isolates CA-1, CA-2, CA-6, F-9, and F-10; urinary, for isolates CA-3, CA-5, F-8, F-9, IV-11, IV-13, and IV-14; blood, for IV-12; and bone for CA-4. Isolates were collected from 1996 to 2002 in 4 of the 7 departments in which the pig farmers were working and were kindly provided by state veterinary laboratories. All strains had been identified with conventional techniques, and their susceptibility to antimicrobial agents had been determined by the disk-diffusion technique (available from www.sfm.asso.fr).

*S. aureus* strains were lysed with 30 μg/mL lysostaphin, which was incubated for 10 min at 37°C, and DNA was extracted by using MagNA Pure LC automat (Roche, Mannheim, Germany), as recommended by the manufacturer. DNA concentrations were measured by optical density, and extracts were diluted to obtain concentrations of 50 ng/μL DNA for amplification.

The presence of *mecA* and *nuc* genes was determined by multiplex polymerase chain reaction (PCR) using *mecA1*, *mecA2*, *nuc1,* and *nuc2* primers (6). Mixes contained 250 μmol/L of each primer, 400 nmol/L of each deoxynucleoside triphosphate (Boehringer GmbH, Mannheim, Germany), 1 × reaction buffer supplied by the manufacturer with 1.5 mmol MgCl_2_, 1 U of AmpliTaq DNA polymerase (Applera, Courtaboeuf, France), and 100 ng of DNA extract in a final volume of 50 μL. The PCR was carried out for 1 cycle of 5 min denaturation at 94°C and 20 cycles of 10 s at 94°C, 10 s at 60°C, and 30 s at 72°C. PCR products were visualized under UV irradiation after electrophoresis.

MLST analysis was carried out by sequencing fragments of 7 housekeeping genes (*arcC*, *aroE*, *glpF*, *gmk*, *pta*, *tpi*, and *yqiL*), as described (available form www.mlst.net), except that the primers used for *tpi* amplification were tpi2u 5´-GCATTAGCAGATTTAGGCGTTA-3´ and tpi2d 5´-TGCACCTTCTAACAATTGTACGA-3´. All PCR products were purified by using the QIAquick PCR purification kit (Qiagen, Courtaboeuf, France) and sequenced using an ABI Prism sequence (Applera) with Big Dye reaction mixes, using the primers chosen for the initial amplification, and analyzed on the BioEdit biological sequence editor 5.0.6 (7). Each allele of the 7 housekeeping genes was assigned to a number, and each isolate was characterized by a sequence type (ST), defined by the allelic profile of the housekeeping genes. These profiles were compared to those present in the *S. aureus* MLST database (available from www.mlst.net). The 2 new allele sequences of the *yqiL* gene and the 1 new sequence of the *aroE* gene were deposited in the MLST database under numbers 72, 73, and 91, respectively. The new STs have also been deposited in the MLST database, under numbers ST432 to ST438, ST440, and ST457.

For each strain, the sequences of all 7 housekeeping genes were concatenated to produce an in-frame sequence of 3,198 bp. A phylogenetic tree (Figure) was generated by using the neighbor-joining method, and the robustness of branches was estimated by the bootstrap method. Both are included in Mega version 2.1 software (available from www.megasoftware.net).

**Figure:**
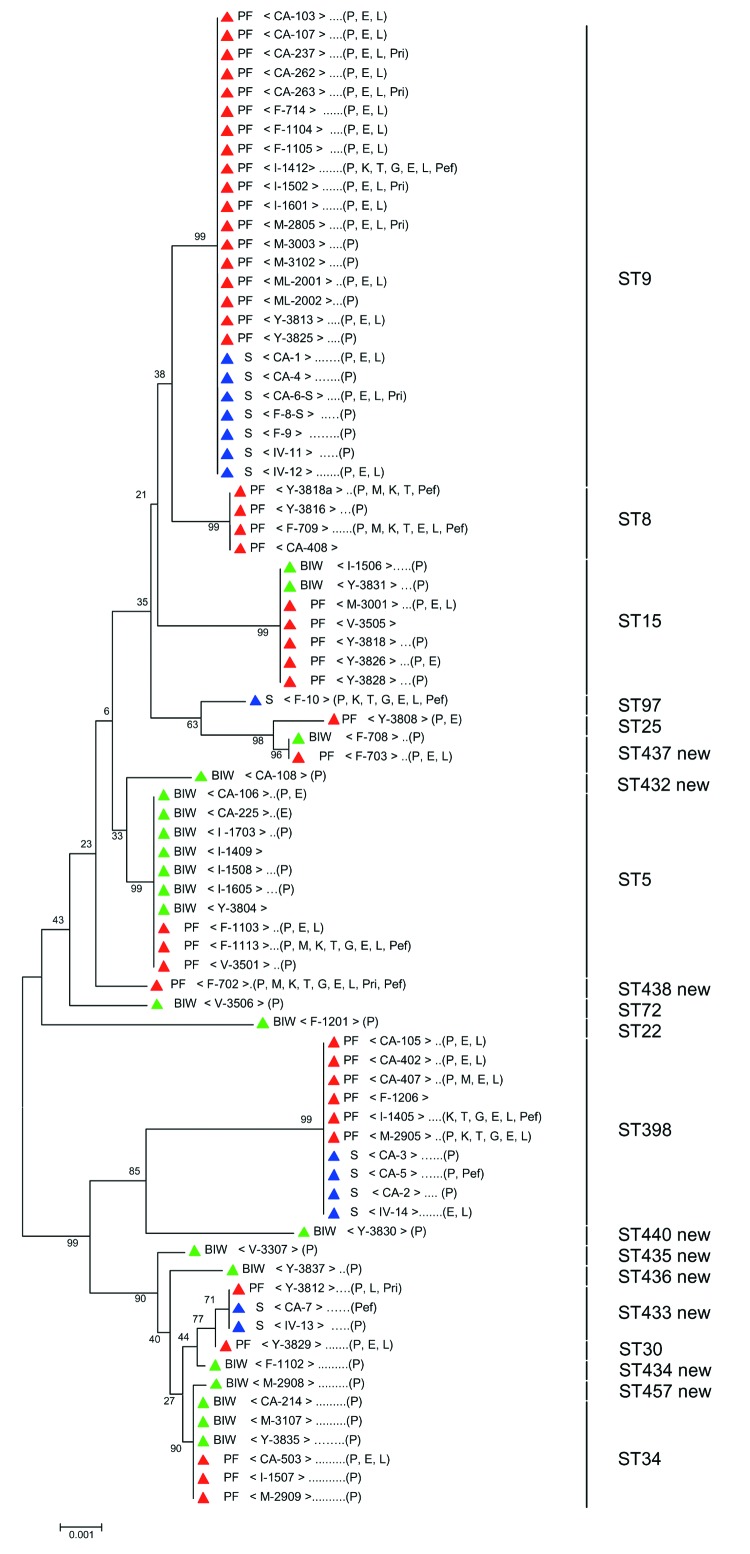
Unrooted tree showing the phylogenetic relationships among Staphylococcus aureus isolates from pig farmers (PF), bank or insurance workers (BIW), and swine (S). The tree was obtained by the neighbor-joining method, based on the comparison of partial sequences of 7 housekeeping genes (arcC, aroE, glpF, gmk, pta, tpi, and yqiL). Values (in percentages) above the lines indicate how the tree's branches are supported by the results of bootstrap analysis. Scale bar = accumulated changes per nucleotide. Isolates from PF, BIW, and S are indicated by red, green, and blue triangles, respectively. Letters between square brackets indicate departments where strains were isolated (CA, Côte d'Armor; F, Finistère; IV, Ile et vilaine; M, Morbihan; ML, Maine et Loire;V, Vendée;Y, Yonne). Letters in parenthesis indicate the antimicrobial agents to which strains were resistant (E, erythromycin; G, gentamicin; K, kanamycin; L, lincomycin; M, methicillin; P, penicillin; Pef, pefloxacin; Pri, pristinamycin; and T, tobramycin). ST, sequence type. ST numbers shown on the right of the tree are from the S. aureus multilocus sequence typing database.

All 79 isolates studied were identified as *S. aureus* by conventional techniques and harbored the *nuc* gene. The *mecA* gene was present in the 5 methicillin-resistant isolates. The Figure shows an unrooted tree in which the aligned sequences of the 79 isolates are compared; it also indicate the ST number and antimicrobial resistance of each isolate.

Nineteen STs were identified among the 65 nasal isolates from pig farmers and nonfarmer controls. Nine (STs 432 to 438, ST440, and ST457) had not been previously described. Twelve of the 19 STs were each found in only 1 isolate, 1 (ST 437) in 2 isolates, and the remaining 6 (ST5, ST8, ST9, ST15, ST34, and ST398) in at least 4 isolates. Only 3 of these 6 STs (ST5, ST15, and ST34) were found in isolates from both pig farmers and nonfarmer controls. ST5 was present in 10 isolates (7 from farmers, 3 from controls), ST15 in 7 (5 from farmers, 2 from controls), and ST34 in 6 (3 from farmers, 3 from controls). Comparison with isolates from the entire MLST database showed that ST5 had previously been reported in 90 isolates from the United Kingdom, Japan, United States, and Poland; ST15 in 33 isolates from the United Kingdom, Australia, and Canada; and ST34 in 15 isolates from the United Kingdom only. The other 3 STs (STs 8, 9, and 398) were only found in isolates from pig farmers. ST8, retrieved from 4 isolates from pig farmers, had previously been reported in 86 isolates from the United Kingdom, Australia, United States, Canada, France, Germany, Netherlands, Denmark, and Greece. ST9 was found in as many as 18 of the 44 pig farmer isolates that we studied but had only been previously described in 5 isolates, all from the United Kingdom. ST398 was retrieved from 6 isolates from pig farmers; previously, it had only been reported in 1 isolate from the Netherlands.

Analysis of the geographic distribution of STs 8, 9, and 398, which were only found in pig farmers, showed that they were dispersed throughout the 7 departments studied. The 18 ST9 isolates were from pig farmers working in 6 of the 7 departments, the 4 ST8 isolates from pig farmers in 3 of 7, and the 6 ST398 isolates from pig farmers in 4 of 7 departments.

Thirteen of the 14 isolates from swine infections had STs that were only found elsewhere in strains from pig farmers. Two of these 13 swine isolates had ST433, which we found in a single pig farmer isolate, 7 had ST9, and 4 ST398 (Figure). STs9, 398, and 433 in the swine isolates originated from 3, 2, and 2 different departments, respectively. The remaining swine isolate had ST97, which was not observed in another isolate. In all, 25 (57%) of the 44 pig farmers isolates had STs identical to those of swine strains. No control isolate was identical to those of the swine.

Four of the 5 methicillin-resistant *S. aureus* (MRSA) strains found in pig farmer isolates had STs (ST5 and ST8) previously reported in MRSA (available from www.mlst.net) or new (ST438). The remaining strain had ST398, which was grouped together with pig farmer isolates that were susceptible to methicillin. Differences in susceptibility to antimicrobial agents other than methicillin were also observed between isolates with identical STs. Although 25 of 25 isolates with ST9 were resistant to penicillin, only 17 were resistant to lincomycin and erythromycin. Of the latter, 5 were coresistant to pristinamycin. One was resistant to kanamycin and pefloxacin. Similar variations in antimicrobial susceptibility were observed among strains with the other STs. Resistance to erythromycin was more frequent in pig farmers than controls (29/44 [66%] vs. 2/21 [10%], as previously reported (4). Resistance was intermediate in swine strains (5 [38%] of 14).

## Conclusions

Our results strongly suggest that the high risk for nasal *S. aureus* colonization that we previously reported in pig farmers (4) was due to strains exchanged with swine: 25 (57%) of the 44 pig farmer isolates grouped together with the swine isolates and had 3 STs (9, 398, and 433) that were not found in control isolates. If these pig farmers had not been taken into account, the rate of nasal carriage in pig farmers would have been close to that found in controls (5). The number of pig strains tested was small because swine mastitis, unlike bovine mastitis, is rare and because no other collection of *S. aureus* from swine was available for testing (we did not sample pigs when we performed the previous (4) study of pig farmers and controls).

The hypothesis that pig farmers exchanged strains bearing these specific STs with swine was not formally demonstrated in our study because we did not test strains isolated from swine and from farmers on the same farms at the same time. However, MLST is a powerful tool for comparing strains and determining their phylogenetic and epidemiologic relatedness (8). This tool, together with the similarity of the pig farmer and swine strains (which both had STs 9, 398, and 433) and the absence of these STs in strains from epidemiologically matched controls, argues strongly in support of an exchange of specific strains between pig farmers and pigs.

The widespread geographic distribution of strains with similar STs in strains in pig farmers and swine only was puzzling because pig farmers were working in different farms scattered among the 7 major French pig-raising departments and separated by tens or sometimes hundreds of kilometers. The potential sources of contamination of pigs and farmers by strains such as those with STs 9, 398, or 433, which were common to both, were not investigated. One possibility might be that the sows used for pig production are the vectors of transmission because young sows are transferred from 1 farm to another when production needs to be increased. A specific investigation is needed to explore this possibility. Contamination of 9 sheep farms and their dairy products by a single *S. aureus* strain was previously demonstrated in France, but the routes of dissemination were not investigated (9). In California, the widespread distribution of a multidrug-resistant clone of *Escherichia coli* that caused community-acquired urinary tract infections was suspected to be of animal origin, but again, the routes of dissemination were not investigated (10).

We observed differences in susceptibility to antimicrobial agents between strains with the same ST, which suggests that the final phenotypes were selected locally, depending on the use of antimicrobial agents. This finding was particularly striking with susceptibility to macrolides and related drugs, a class of antimicrobial agent widely used in pig farming (11), although individual farms may use it differently. Unfortunately, data on the use of antimicrobial agents by each farm were not available.

Animal-to-human transmission during farming has been demonstrated for enterobacteria and enterococci in several instances (1,2) but only once before for *S. aureus* (9). Although they are rare (12), animal MRSA have been suggested as a source of infection for humans (13). Our results suggest that such transmission may be frequent, particularly since virtually no barrier precautions were used by the pig farmers studied in our previous investigation (4).

In the few published studies on the molecular epidemiology of nasal strains from carriers, genetic backgrounds of the strains were very diverse (14), just as in our controls. This finding further underlines the particular way in which most pig farmer strains are grouped together. Whatever the exact route of transmission, single *S. aureus* strains, probably acquired from pigs, colonized the nostrils of pig farmers throughout large geographic areas and that this colonization probably caused their overall increase in nasal *S. aureus* colonization. Since nasal carriage is a recognized source of *S. aureus* bacteremia with severe consequences (15), our findings suggest that pig farming could be a staphylococcal hazard for farmers, under the conditions in which it is practiced today. Several points could not be addressed in the study, including whether colonization of the farmers by pig *S. aureus* isolates was permanent or temporary, whether the pig isolates were also disseminated in the farmer's families and to other persons living in the area, whether skin and soft tissues of pig farmers were infected and, if so, whether or not it was due to *S. aureus* isolates identical to those from pigs. These questions will be addressed in further, specifically designed, epidemiologic studies.
